# Evolution of swallowing in lateral pharyngoplasty with stylopharyngeal muscle preservation

**DOI:** 10.5935/1808-8694.20120033

**Published:** 2015-10-20

**Authors:** Jayson Mesti, Michel Burihan Cahali

**Affiliations:** a MD. Otorhinolaryngologist; b PhD in Sciences - School of Medicine of the University of São Paulo. Coordinator of the Sleep Medicine Ward of the Otorhinolaryngology Department - Hospital do Servidor Público Estadual de São Paulo

**Keywords:** deglutition, deglutition disorders, snoring

## Abstract

Lateral pharyngoplasty manages obstructive sleep apnea through the myotomy and repositioning of the muscles of the lateral pharyngeal wall. Dysphagia after any pharyngeal surgery is influenced by pain, discomfort from the sutures, the healing process and by the adaptation to the changes in pharyngeal structures. Experience with lateral pharyngoplasty has shown that the superior pharyngeal constrictor muscle plays a minor role in swallowing. One of them, the stylopharyngeus muscle, seems to play an important role during swallowing.

**Objective:**

The aim of this study is to provide a daily analysis of the follow-up of the swallowing function.

**Method:**

We have prospectively evaluated the swallowing function in 20 patients, through the daily application of a visual analogue scale from the first post-op until the complete disappearance of dysphagia.

**Results:**

Patients have returned to their normal feeding habits in a mean of 10.9 days after the procedures and they presented a completely normal swallowing, on average, 21.6 days after the surgeries. All patients recover normal swallowing after the procedures, with a maximum recovery time of 33 days.

**Conclusion:**

In this study, all patients who underwent lateral pharyngoplasty with total preservation of the stylopharyngeus muscle reported complete normalization of swallowing with a recovery time up to 33 days.

## INTRODUCTION

The lateral pharyngeal wall of patients with obstructive sleep apnea (OSA) has a greater tendency to collapse during the passage of air flow when compared to patients without OSA^1^, ^2^, very likely because of a delay in the relaxation of the constrictor muscles on the expiratory-inspiratory transition^3^. The lateral pharyngoplasty (LP) was developed in order to act on these pathophysiological aspects of OSA. LP is based on reconstructing the pharyngeal lateral wall, by the myotomy of its superior constrictor muscles and suturing of the laterally pedicle flaps to the palatoglossus muscle^4^. In its second version, from 2004^5^, the LP no longer included uvulectomy, palatopharyngeal zetaplasty and the need to use the surgical microscope. This approach removes the constricting tension of the lateral pharyngeal walls, enabling its lateral expansion. Nonetheless, there are other muscles on this wall participating in the pharyngeal functions.

The pharyngeal muscles have two layers: the most external, cross-sectional and “U-shaped”, made up by three pairs of constrictor muscles (superior, middle and inferior), which act on pushing food to the esophagus by means of sequential involuntary contraction; the innermost layer, formed by three pairs of longitudinal muscles, stemming from the styloid process, cartilaginous portion of the auditory tube and soft palate^6^. These are, respective, the stylopharyngeus, salpingopharyngeus and palatopharyngeal muscles, responsible for elevating the pharynx during deglutition^6^. Functionally speaking, the pharyngeal shortening increases the pushing force by reducing pharyngeal volume^7^. Anatomically speaking, the longitudinal pharyngeal muscles are located on the lateral pharyngeal wall and, inferiorly, they are inserted on the posterior border of the thyroid cartilage^7^, ^8^.

The stylopharyngeus, one of the muscles responsible for the pharyngeal phase of swallowing, it is a tapered muscle, slender and long, which descends between the external and internal carotid arteries, and it penetrates the pharyngeal wall between the superior and middle constrictor muscles, running longitudinally and deep in relation to the superior constrictor muscle and superficially in relation to the middle constrictor. On the lateral pharyngeal wall, the stylopharyngeus is anterior to the buccopharyngeal fascia and posterior to the superior constrictor muscle, and it is innervated by the glossopharyngeal nerve^7^, ^8^. This muscle raises the pharynx, compressing the lateral laryngeal walls and helping in the pharyngeal compression over the food bolus during deglutition. Since otorhinolaryngologists are not very familiarized with this anatomy, the myotomy of the superior constrictor muscle of the pharynx, especially in its inferior portion, they may also cut part of the stylopharyngeus and fibers of the medium constrictor muscle.

Starting in 2008, the second author of the present study included the systematic identification and preservation of the stylopharyngeus muscles in the lateral pharyngoplasty approach. The goal of the present study is to assess the daily evolution of postoperative deglutition after lateral pharyngoplasty done with this innovative technique. According to our knowledge, there are no reports of daily assessments on the deglutition of patients submitted to surgery to treat OSA.

## METHOD

We ran a prospective study involving 20 adult patients (older than 18 years), diagnosed with OSA (hypopnea-apnea index of 5 - HAI3 5), submitted to lateral pharyngoplasty to treat this disorder. All the patients were informed about non-surgical treatments for OSA, such as CPAP and intraoral device, according to the indication in each case, and they refused these. All the patients were submitted to a complete otorhinolaryngology exam, full night assisted polysomnographic test in a sleep lab and the usual preoperative tests required for surgeries under general anesthesia. We included in the study, patients from both genders, without prior pharyngeal surgeries. The use of a dental prosthesis was not a reason for exclusion. We took off the study those patients who complained of dysphagia in the preoperative, those with high body-mass index (BMI), greater than 35 kg/m^2^, patients using benzodiazepine agents or other drugs which depress the central nervous system and patients with anesthetic risk III or IV in the ASA. This research protocol was approved by the Ethics Committee of our institution, under number 116/08, and all the subjects in the study signed the free and informed consent form.

### Surgical technique

On the lateral pharyngoplasty utilized in this study^5^, we initially did tonsillectomy in one of the sides and, following that, we removed a triangle of mucosa, together with some fibers of the palatoglossus muscle, the palatine corner, in order to broaden the exposure of the lateral wall and that of the superior constrictor muscle of the pharynx. The size of the triangle removed depends on the stretching of the posterior pillar, which with little tensioning, must cover the resected area at the end of the procedure. Once broadly exposed, we detach and raise the superior constrictor muscle from the buccopharyngeal fascia, which is located behind this muscle.

**Figure 1:**
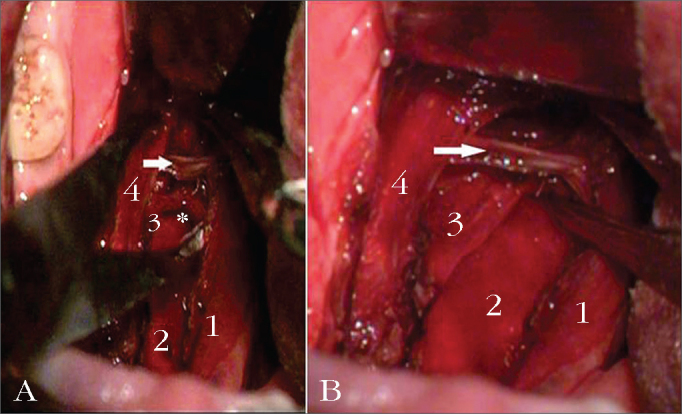
A: (without medial traction of the palatopharyngeal. Intraoperative aspect of the lateral pharyngoplasty with preservation of the stylopharyngeus muscle. 1: palatopharyngeal m.; 2: buccopharyngeal fascia; 3: stylopharyngeus m.; 4: palatoglossus m.; * crossover of the stylopharyngeus m. with the middle constrictor; (arrow) superior constrictor m; B: (with medial traction of the pharyngeal palate). Intraoperative view of the lateral pharyngoplasty, preserving the stylopharyngeus muscle. 1: palatopharyngeal m.; 2: buccopharyngeal fascia; 3: stylopharyngeus m.; 4: palatoglossus m.; * crossover of the stylopharyngeus m. with the middle constrictor; (arrow) superior constrictor m.

Following that, we carry out the myotomy of the constrictor at the posterior wall of the pharynx, in the cranial-caudal direction, after cauterizing its fibers with the bipolar scalpel. The constrictor muscle detachment and myotomy start in its most cranial portion, just above the uvula implantation site, and always near the posterior tonsil pillar. At the lower third of the tonsillar fossa, we isolated the stylopharyngeus muscle from the constrictor, and we did the myotomy to the constrictor inferiorly all the way to its lingual insertion, preserving the stylopharyngeus muscle. It is easier to identify the stylopharyngeus when we pull the palatopharyngeal muscle medially, medially shifting the muscles on the lateral wall (Figure 1A-B). The lateral part of the constrictor is then sutured to the anterior pillar with separated stitches, using 4.0 Vicryl® (polyglactin 910, Ethicon®). This line of suture repositions the lateral constrictor flap and improves local hemostasis. The exposed peripharyngeal space, without constrictor muscle tension and with a supported lateral wall, is then closed by approximation and suture of the posterior pillar to the anterior pillar. The medial portion of the cut constrictor is not sutured. All the steps are repeated on the contralateral side and the uvula is fully preserved.

### Daily assessment of deglutition

All the patients filled out a subjective questionnaire of deglutition on a daily basis. The filling out started on the first day of postoperative, always after lunch, when the patients assigned a score to their “difficulty to swallow”, between 0 and 10, 0 representing no difficulty and 10 representing the highest difficulty. Filling out the questionnaire continued until complete remission of any difficulty to swallow, in other words, until the patient reached the score zero. Each patient received a numbered questionnaire form per day, and they returned it to the examiner when they returned for their regular follow up visits. Moreover, the patients made a summary report of the content of their lunch meals, everyday.

We also analyzed the time, in days, in which the patients reported having returned to their regular diets and the time in days until the patients reached the score 0, according to the questionnaire form.

## RESULTS

**Graph 1:**
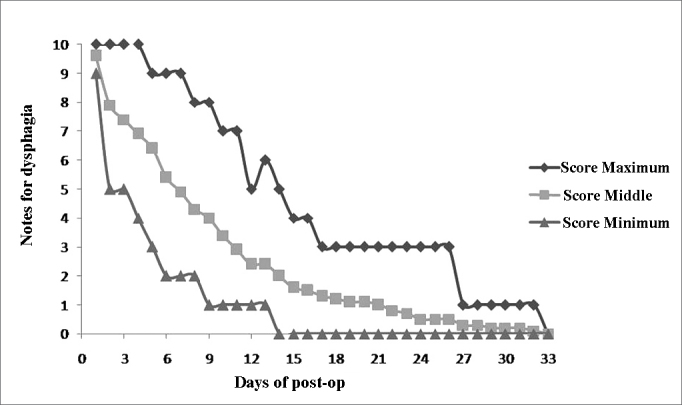
Assessing the deglutition difficulty after lateral pharyngoplasty, with the maximum (upper curve), minimum (lower curve) and middle (middle curve) scores assigned by the 20 patients who were assessed in a daily basis.

The 20 patients were assessed between March of 2008 and August of 2009. The series was made up of 15 men (75%) and five women (25%). The mean age of the group was 45.7 years and the mean HAI was 23.8. Six patients (30%) had severe OSA (HAI > 30), seven patients (35%) had moderate OSA (15 ≤ IAH ≤ 30) and seven patients (35%) had mild OSA (5 ≤ IAH < 15). The daily development of postoperative deglutition was plotted on Graph 1. The patients reported returning to their regular diets between the 5^th^ and 17^th^ days - 10.9 days after surgery, in average. Fifty percent of the cases returned to their regular diets by the 10^th^ day of post-op.

The patients reported having totally normal deglutition (difficulty 0) in an average of 21.6 days after surgery, ranging between 14 and 33 days.

## DISCUSSION

The surgical handling of muscle layers on the lateral pharyngeal wall to treat OSA is somewhat recent in the history of surgery^3^ and detailed data on the evolution of deglutition facing these muscle reconstructions are important in the preoperative education of patients. Lateral pharyngoplasty is a technique which has been increasingly used in the treatment of OSA, since it brings about better clinical and polysomnographic results when compared to uvulopalatopharyngoplasty (UPPP)^9^. We do not know of any similar study in the literature concerning the outcome of swallowing in the different surgeries performed to treat OSA.

UPPP is one the most common procedures used to treat OSA, described by Fujita in the west, in 1981^10^. Some of its complications are transient and very common, such as bleeding, velopharyngeal insufficiency and dysphagia^11^, ^12^. It seems that dysphagia after UPPP happens due to a different mechanism from the one caused by the LP. In the UPPP, there probably is a deterioration on local sensitivity because of the incision on the tissues of the soft palate and the tonsil pillars, and also, due to the pharyngeal fibrosis stemming from the tension of the sutures on the pillars, and a possible velopharyngeal insufficiency because of the resection done on the central region of the soft palate^11^, ^12^. This technique is based on the concept of maximum removal of the pharyngeal mucosa with preservation of its muscles^10^. The pathophysiological understanding of the OSA is currently being developed, guiding the evolution of surgical treatment in this field, with significant changes in the concepts created by the UPPP^13^, ^14^, ^15^, ^16^, ^17^.

In LP, there is a myotomy of the constrictor muscles, which act on the transportation of the food bolus, participating in the shortening of the pharynx upon swallowing, and being responsible for pushing the food to the esophagus, acting from an involuntary and sequential contraction. Functionally, the pharyngeal shortening generates a push force by reducing the pharyngeal volume^7^. LP could change this entire laryngeal mechanics. Notwithstanding, we have noticed that sectioning the superior constrictor causes transient and not very relevant changes, and the patients always return to their regular feeding. In the initial cases involving this approach, on the first version described, with the myotomy of the constrictor together with the insertion of the stylopharyngeus and fibers of the middle constrictor, we have found a substantial variability in the time for complete recovery, ranging between 7 and 70 days^9^. With the identification and preservation of the stylopharyngeus, we noticed a greater regularity in the evolution of deglutition, and dysphagia was transient in all the assessed patients. After 13 years of experience with LP, the second author observes that there is no long term complaint of dysphagia, between 2 and 11 years of follow up, both in young and older patients, those above 65 years of age, contrary to what happens after UPPP, in which some long term dysphagia is not uncommon^11^. We have performed LP as a routine in our service, since 2006, which was when we completely abandoned UPPP.

The work of improving the LP was based on making the postoperative recovery time of dysphagia into the shortest possible. We believe that the function of the superior constrictor is totally compensated by the action of the tongue base on a palate veil that is active and fully preserved in its medial region. Moreover, the longitudinal muscles, the stylopharyngeus among them, which raise the pharynx during deglutition, seem to have a role of fine modulation on the oropharyngeal phase, enabling a relatively fast adaptation of the pharynx to the new positioning of the muscles after LP. Only objective studies on deglutition could be able to confirm these hypothesis. In order to assess swallowing and pain, studies in the literature have routinely used visual-analogue scales, which produce subjective assessments^18^, ^19^, ^20^ with scientific validity. Our study has some limitations. The lack of a control group naturally limits the very relevance of these findings. It is clear that it is utopian to expect that all the changes in details in surgical techniques are presented in agreement to evidence-based medicine. In addition, we did not carry out objective analysis of the deglutition, which could show the impact of LP on the subclinical alterations, since more than half of the patients with OSA have subclinical changes in their swallowing^21^. In the case of LP, we noticed that the preservation of the stylopharyngeus, of the middle constrictor, and all the central area of the soft palate, besides the lack of tension on the sutures made to the pillars, are important factors to accelerate recover of postoperative dysphagia. Our data indicated that, in patients without prior complaints of dysphagia submitted to LP, there is a return to regular diet, in an average of 11 days and 100% of the cases will return to normality in deglutition in a little over one month.

## CONCLUSION

The daily assessment of deglutition after LP, carried out with complete myotomy of the superior constrictor muscle of the pharynx and total preservation of the stylopharyngeus muscle, showed that the patients reported 100% of return to normal deglutition in up to 33 days. A return to regular diet happens, in average in 10.9 days after the surgery, ranging between 5 and 17 days.
